# Tobacco smoking in a high-IQ society in Indonesia

**DOI:** 10.18332/tid/205841

**Published:** 2025-07-23

**Authors:** Teuku Muhammad Haykal Putra, Reynold Siburian, Syahniar Mukmina, Natalia Wardhani, Faris Ervandi Alam, Wittawat Wattanasiriporn, Haris Munirwan

**Affiliations:** 1 Department of Cardiology and Vascular Medicine, Jakarta Heart Center, Jakarta, Indonesia; 2 Department of Obstetrics and Gynecology, Harapan Kita National Women and Children Health Center, Jakarta, Indonesia; 3 MENSA Indonesia, Jakarta, Indonesia; 4 College of Medicine, Rangsit University, Pathum Thani, Thailand; 5 Faculty of Medicine, Universitas Syiah Kuala, Banda Aceh, Indonesia

**Keywords:** smoking, prevalence, IQ, intelligence, epidemiology

## Abstract

**INTRODUCTION:**

Individuals with higher intelligence are often believed to be more aware of the health risks associated with smoking and more inclined to adopt healthier lifestyles. This study investigates the prevalence of smoking among high-IQ individuals in Indonesia, providing insights that could inform targeted health interventions.

**METHODS:**

This is a cross-sectional study conducted in a population with high IQ. An online-based survey form was distributed to all active members of the Mensa high-IQ society in Indonesia over a three-month period (October to December 2024). The survey form collected sociodemographic data and queried participants about their smoking status and related details. The primary outcome of interest was the smoking habits of the subjects.

**RESULTS:**

A total of 102 respondents aged ≥18 years participated in the study, which involved 73 men (71.6%) and 29 women (28.4%), with a mean age of 33.8 ± 10.2 years. The overall smoking prevalence among high-IQ individuals in Indonesia was 10.8%, with 12.3% of men and 6.9% of women currently smoking. Additionally, 9.8% were former smokers, leading to a total current or past smoking rate of 20.6%. This number is dramatically lower than the national smoking prevalence, which currently stands at around 40.3%. Although various sociodemographic factors were assessed, none showed a statistically significant association with smoking status in this population.

**CONCLUSIONS:**

The prevalence of tobacco smoking among high-IQ individuals in Indonesia is 10.8% which is significantly lower than the national average. Although no statistically significant associations were found between sociodemographic variables and smoking status, smokers tended to have a family member who smoked, and individuals with higher annual incomes were less likely to smoke.

## INTRODUCTION

Indonesia stands out for its remarkably high prevalence of tobacco smoking, even compared to its neighbors^1-3^. A national survey published in 2020 revealed that 40.3% of the population were regular smokers, significantly higher than the global average of around 25% for males and 5.4% for females^3,4^. Despite numerous efforts by the government and various community groups, the smoking rate continues to rise^4,5^. Contrary to the global trends where smoking rates are declining, Indonesia saw an increase from 30.8% in 2007 to 31.9% in 2014^2,4,5^. The magnitude of this health issue is alarming, considering the associated risk of many serious diseases including cardiovascular disease, lung disease, and cancer^4^.

The association between intelligence and smoking has been hypothesized since the CARDIA study back in 1990. It revealed a strong inverse relationship between education level and smoking prevalence^6^. Investigating the specific link between intelligence and smoking poses significant challenges due to the extensive resources required for widespread IQ testing^7^. Nevertheless, some studies have explored this area of interest^7,8^. An early survey on smoking habits in public high schools revealed that the mean intelligence levels of children who did not smoke were higher than those who did^8^. Recent studies reinforce this finding, showing that smokers generally have lower IQ scores compared to non-smokers, with averages of 95.0 and 100.7, respectively^9,10^. Furthermore, the studies showed that the more cigarettes a person smokes, the lower their cognitive test scores tend to be, and the more rapidly their memory declines^8,9,11^. Additionally, another publication described that lower IQ performance during teenage years is associated with a higher likelihood of smoking in later life^12^. Significant positive effects of intelligence are evident not only in smoking prevalence but also in the likelihood of successful smoking cessation^5,6,13^.

Tobacco smoking in high-IQ societies presents an interesting paradox. People with higher levels of intelligence are associated with better decision-making and allegedly more aware of the health risks associated with smoking, thereby choosing healthier lifestyles^7,13^. Smoking behavior may still be influenced by various factors. These include cognitive awareness and risk aversion, social and cultural influences, stress, anxiety coping mechanism, personality traits, and substance use^9^. Smoking rate among high-IQ individuals was first documented through a quick survey conducted in Australia in 1985. The results supported the proposition that the prevalence of smoking was significantly below the national average^7^. A more recent study from Greece in 2021 explored smoking behavior in gifted individuals similarly concluded that this population is less likely to smoke. However, the term ‘gifted’ is broad and contentious and may only partially overlap with the construct of high IQ in its spectrum. Moreover, this particular study relied on self-identification to classify individuals as gifted, raising concerns about the validity of the high IQ in that context^14^. No similar studies have been published in any other parts of the world since then. Additionally, sociodemographic data on high-IQ individuals could provide valuable insights to explore effective smoking cessation programs. This research aims to explore the prevalence of smoking among the high-IQ population in Indonesia, offering a new perspective on targeted health interventions.

## METHODS

### Study design and sample

This is a cross-sectional study conducted specifically in a population of high-IQ society in Indonesia. Mensa is the largest and the oldest high-IQ society in the world. It is a non-profit organization that includes individuals who score at or above the 98th percentile on a standardized IQ test. Founded in 1946, Mensa has established national groups across the world, including Mensa Indonesia, which has been active since 1991. By the time this study was conducted, there were 408 active members of Mensa Indonesia.

### Online survey

An online-based survey form was personally distributed via message to all active members of Mensa Indonesia over a three-month period (October to December 2024). All members had an equal opportunity to participate by simply completing the online survey. Follow-up reminders were sent via personal messages twice a month throughout the sampling period. No randomization techniques were employed in order to maximize the number of responders. The survey form included an informed consent and collected sociodemographic data. Ethnic groups were classified accordingly^15^. It also queried participants about their smoking status and related details, which they were to fill in voluntarily. The primary outcome of interest was the smoking habits of the subjects. The survey also explored detail aspects of smoking behavior including participants’ awareness of smoking-related health risks, the presence of current smokers in the family, the type of tobacco products used, the number of cigarettes consumed daily, and the estimated monthly expenditure on smoking. Participants were classified as current smokers if they reported regular smoking or if they had smoked at least one cigarette in the last 30 days^16^. The survey was conducted bilingually, in English and Bahasa Indonesia, to accommodate all participants. Finally, the survey yielded responses from 102 out of 408 active members of Mensa Indonesia, resulting in a response rate of 25%. Ethical clearance for the study was obtained from the Institutional Review Board at the Jakarta Heart Center, Indonesia. Written informed consent was collected from all the subjects.

### Statistical analysis

Continuous variables that are normally distributed are presented as mean ± standard deviation (SD), while non-normally distributed continuous variables are shown as median and interquartile range (IQR). The independent Student’s t-test was utilized to analyze differences in means between two groups. For differences in medians, the Mann-Whitney U test was employed. Categorical data are given as frequencies and percentages, with differences analyzed using the chi-squared test and Fisher’s exact test, as appropriate. Both univariate and multivariable logistic regression models were used for analysis. Variables from the univariate model with a p-value of less than 0.25 were included in the multivariable analysis^17^. Adjusted odds ratio (AOR) and 95% confidence intervals are calculated and presented. All statistical analyses were performed using IBM SPSS Statistics 20 (SPSS Inc., Chicago, USA). A p<0.05 was considered statistically significant.

## RESULTS

The online questionnaire was completed by 102 subjects. This study ultimately involved 73 men (71.6%) and 29 women (28.4%), with a mean age of 33.8 ± 10.2 years. There are 26 (25.5%) obese participants in the study. The ethnic composition of the study group was predominantly Indonesian Chinese (40.2%) and Javanese (20.6%). Forty-seven subjects were married, while the remaining 55 (52.9%) subjects were single. Other sociodemographic data of the study are presented in Table 1.

**Table 1:** Baseline characteristics of respondents in a 2024 survey of high-IQ society members in Indonesia (N=102)

*Characteristics*	*n (%)*
**Gender**	
Male	73 (71.6)
Female	29 (28.4)
**Age** (years), mean ± SD	33.8 ± 10.2
**BMI** (kg/m^2^), mean ± SD	24.9 ± 4.2
**BMI classification**	
Underweight	4 (3.9)
Normal	31 (30.4)
Overweight	41 (40.2)
Obese	26 (25.5)
**Ethnic group**	
Javanese	21 (20.6)
Sundanese	5 (4.9)
Chinese	41 (40.2)
Melayu	5 (4.9)
Other	30 (29.4)
**Marital status**	
Married	48 (47.1)
Divorced/widowed/separated	3 (2.9)
Never married	51 (50.0)
**Education level**	
High school or lower	9 (8.8)
Bachelors/Diploma (S1/D1-4)	49 (48.0)
Master (S2/Specialist)	39 (38.2)
PhD (S3/Consultants)	5 (4.9)
**Profession**	
Business owner	16 (15.7)
Professional	25 (24.5)
Student	4 (3.9)
Employee	46 (45.1)
Other	11 (10.8)
**Regular coffee consumption**	53 (52.0)
**Annual income** (US$)	
<3750	16 (15.7)
3751–10000	27 (26.5)
10001–25000	26 (25.5)
25001–50000	15 (14.7)
50001–100000	10 (9.8)
>100000	8 (7.8)

BMI: body mass index.

The prevalence of smoking among members of Mensa Indonesia stands at 10.8%. There was a noticeable difference in smoking rates between men and women, presenting with 12.3% and 6.9%, respectively. However, subsequent bivariate analysis revealed that this difference was not statistically significant. Furthermore, 9.8% of subjects reported being former smokers, bringing the combined rate for current and past smoking to 20.6%. In addition, nearly all participants were aware of the health risks associated with tobacco use.

Among those who are current smokers or have a history of smoking, the median daily cigarette consumption is 8, with a range from 1 to 32 cigarettes in a day. The estimated monthly cost of smoking was also explored, with the median cost being US$8 (range: 1–62) . Other details on the smoking habits of the subjects are presented in Table 2.

**Table 2:** Smoking status of respondents in a 2024 survey of high-IQ society members in Indonesia (N=102)

*Smoking status*	*n (%)*
**Tobacco smoking**	
Current smoker	11 (10.8)
Former smoker	10 (9.8)
Never smoker	81 (79.4)
**Aware of health issues caused by smoking**	101 (99.0)
**Current smoker in the family**	36 (35.3)
**Type of tobacco smoking**	
Filtered	16 (80.0)
Unfiltered	4 (20.0)
**Number of daily cigarettes,** n (IQR)	8 (10)
**Estimated monthly cost for smoking,** US$ (IQR)	12 (25)
**Other types of smoking**	
E-cigarettes	3 (2.9)
Vape	5 (4.9)

IQR: interquartile range.

**Figure 1:**
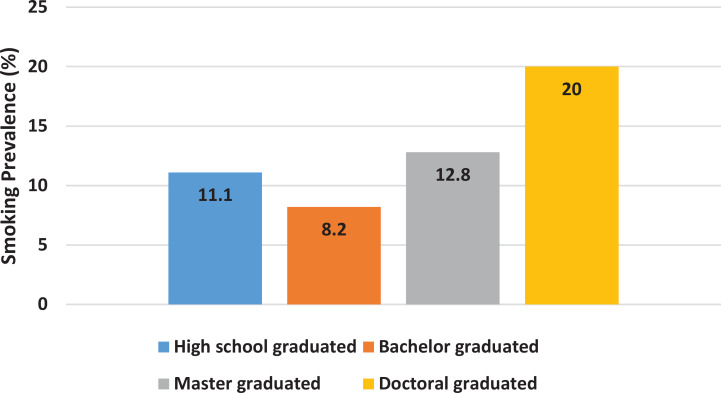
Smoking prevalence by education level in a 2024 survey of high-IQ society members in Indonesia (N=102)

**Figure 2:**
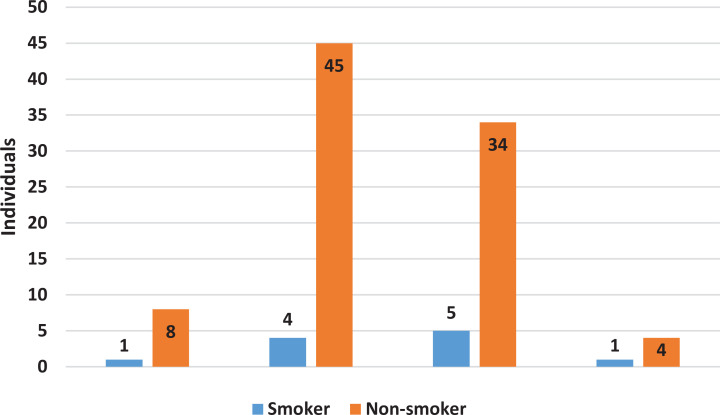
Comparison of smoker and non-smoker by education level in a 2024 survey of high-IQ society members in Indonesia (N=102)

We explored various sociodemographic data and other habits to identify any associations with current smoking status. Bivariate analysis revealed no significant associations with any of the variables. Although education level was not significantly associated with smoking status (p=0.811; Table 3), we included descriptive graphs to illustrate the distribution of smoking prevalence across different levels of education in our study population (Figure 1 and Figure 2). Some variables with conspicuous differences include the presence of a current smoker within the close family and annual income. However, statistical analysis revealed no significant p-values for any associations. We proceeded with multivariable analysis for these two variables, given their p-values were below 0.25. Nevertheless, the results remained consistent, indicating that these two variables are not significantly associated with the smoking status of the subjects (Table 4). The presence of a current smoker in the family accounted for AOR of 2.83 (95% CI: 0.54–14.82, p=0.220), while an annual income >US$25000 yielded an AOR of 0.16 (95% CI: 0.19–1.32, p=0.088).

**Table 3:** Bivariate analysis of smoking status in a 2024 survey of high-IQ society members in Indonesia (N=102)

*Variables*	*Smoker (N=11)* *n (%)*	*Non-Smoker (N=91)* *n (%)*	*p*
**Male**	9 (81.8)	64 (70.3)	0.425^a^
**Age** (years), median (IQR)	33 (18–74)	31 (23–68)	0.582^b^
**BMI** (kg/m^2^), mean ± SD	25.1 ± 4.9	24.9 ± 4.2	0.911^c^
**Obese**	3 (27.3)	23 (25.3)	0.886^a^
**Marital status**			
Married	4 (36.4)	44 (48.4)	0.335^a^
Divorced/widowed/separated/single	7 (63.6)	47 (51.6)	
**Education level**			
High school or lower	1 (9.1)	8 (8.8)	0.811^a^
Bachelors/Diploma (S1/D1-4)	4 (36.4)	45 (49.5)	
Master (S2/Specialist)	5 (45.5)	34 (37.4)	
PhD (S3/Consultants)	1 (9.1)	4 (4.4)	
**Regular coffee consumption**	6 (54.5)	47 (51.6)	0.856^a^
**Current smoker in the family**	2 (18.2)	34 (37.4)	0.180^a^
**Annual income** (US$)			
≤25000	10 (90.9)	60 (65.9)	0.083^a^
>25000	1 (9.1)	31 (34.1)	

a Pearson’s chi-squared test of Fisher’s exact test.

b Mann-Whitney U test.

c Student’s t-test.

**Table 4:** Logistic regression analysis of factors associated with smoking status in a 2024 survey of high-IQ society members in Indonesia (N=102)

*Variables*	*AOR*	*95% CI*	*p*
**Age** (years)	0.99	0.94–1.06	0.932
**Male**	1.72	0.33–9.11	0.523
**Current smoker in the family**	2.83	0.54–14.82	0.220
**Annual income**	0.16	0.19–1.32	0.088

AOR: adjusted odds ratio.

## DISCUSSION

From the very beginning of this research, we anticipated that the smoking prevalence among the high-IQ population in Indonesia would be lower than the national average^7^. Indeed, this study confirmed the hypothesis, identifying a smoking prevalence of 10.8% among the high-IQ population in Indonesia. This number is dramatically lower than the national smoking prevalence, which currently stands at around 40.3%^4^. Based on this comparison, the prevalence of smoking in Indonesian high-IQ population appears to be roughly one-quarter of that in the general population.

It is widely accepted that higher intelligence is negatively associated with smoking habits^6^. Past researches observed lower IQ scores in smokers compared to non-smokers. However, it remains a challenge to ascertain whether smoking prevalence is inherently lower in high-IQ individuals compared to those with lower IQ performance. This question was indirectly addressed by a previous study among Mensa Australia members in 1985. They reported a smoking prevalence of 19.9%, significantly below the national rates of 40% for men and 31% for women at that time^7^. The results of our study similarly demonstrate this phenomenon, showing that the prevalence of tobacco smoking among high-IQ individuals is far below the national smoking prevalence. This finding adds to the observed pattern of lower smoking prevalence among individuals with higher IQ, though the underlying reasons for this association remain to be fully understood. Factors such as better health knowledge and increased awareness of smoking-related risks may contribute to this trend.

Research targeting individuals with high IQ presents unique challenges. The higher the IQ percentile, the more difficult the recruitment becomes. Conducting blinded sampling in the general population and testing everyone’s IQ would be exhaustive and yield only a select few suitable individuals^7^. Our study, aimed specifically at determining the prevalence of smoking among high-IQ individuals, was made feasible through coordination with Mensa Indonesia. Mensa Indonesia began operations in 1991 as an affiliate of Mensa International. They hold routine entrance examinations for individuals who must demonstrate their IQ performance on a standardized test. The test used is a derivative of the Stanford-Binet test, generated by Mensa International to be free of language and national barriers. Admission to the organization is granted if the individual’s test results are at the 98th percentile or above. This corresponds to an IQ score higher than 128–130. The cutoff of 128–130 using a standard deviation of 15 is commonly used to categorize individuals as having high IQ^7^. This organization actively engages its members through an online forum and also conducts routine offline meetings to discuss various topics. However, it is crucial to note that the population of Mensa Indonesia does not necessarily represent all high-IQ individuals in the country. Considering Indonesia’s population exceeds 280 million, it is expected that approximately 5.5 million people would be eligible to be Mensa members. The fact that Mensa Indonesia comprises only around 400 members is partly due to the geographical challenges of the country, which make it difficult to perform routine offline entrance examinations in all regions.

Numerous social and demographic variables are known to be associated with smoking habits^1,2,5,6,18,19^. Gender consistently appears in multiple publications as a factor associated with smoking rates, with males generally having a higher prevalence than females^1,2,15-17^. The 2021 Global Adult Tobacco Survey (GATS) revealed that in Indonesia, 64.7% of men and 2.3% of women are regular smokers^1^. However, in our study, the gender distribution is similar between groups. This finding is particularly noteworthy given that Mensa membership is predominantly male. Typically, in populations with a balanced gender distribution, significant differences between smokers and non-smokers are clearly evident.

Education level is also commonly associated with smoking habits^2,5,6,16,17^. It was identified as early as 1990 in the CARDIA study. The magnitude of the relationship was strong, with more than a four-fold difference between those holding a graduate degree and those with less than a high school diploma^6^. However, we did not observe the same phenomenon in our study population. Notably, the spike in smoking prevalence typically seen in those who did not graduate from high school was absent in our study, as all participants had completed high school^2,6,16^. We observed a graph of smoking prevalence across different education levels that did not reveal any notable patterns like those documented in previous studies^2,6^. It might be inferred that the threshold for higher smoking rates occurs at the high school graduate level^16^. The advancement of modern society has facilitated the spread of information, making it widely known that smoking poses significant health risks^20^. This type of information is readily accessible and generally understood by individuals with at least a high school education, which typically corresponds to a decent level of health literacy regarding hazards of smoking^20-22^. Supporting this, additional data from our study indicate that almost all participants were aware of and believed in the severe health risks associated with smoking.

Variables that showed notable differences between groups included the presence of a current smoker in the family and annual income. However, neither bivariate nor multivariable analysis demonstrated significant statistical differences between these groups. Both variables have been identified as true predictors of smoking habits in various populations^2,23^. The lack of significant statistical findings in our study might be attributed to the limited sample size. The smoking group contained only 11 individuals. Increasing the number of subjects could potentially reveal differences between groups. However, expanding the sample size poses a significant challenge, as the current sample size was the maximum achievable within a 3-month sampling period.

### Implications

The implications of this study for health policy are arguably vague. The prevalence of smoking among high-IQ individuals in our population was already far below the national average^3,4^. In line with the principle of diminishing returns, directing additional resources specifically to this group would likely yield only marginal gains^24,25^. Moreover, our study revealed that 99% of our population understood the risks of smoking, rendering additional health promotion efforts to increase awareness in vain. Anti-smoking campaigns may have greater impact if they focus on populations with lower IQ, where smoking is more common and awareness of its risks is lower.

### Limitations

This study has a few limitations. First, the response rate of less than a third of the target population raises concerns regarding potential selection bias. However, this is actually inevitable. A previous survey targeting a similar population achieved a significantly higher response rate because it consisted of only three questions^5^. Our more comprehensive three-pages online survey form naturally resulted in a lower response rate. Second, this study may not have been adequately powered to detect significant differences between sociodemographic groups or to adjust for potential confounding factors. While we calculated our minimum sample size to be sufficient for determining the prevalence of smoking within this specific population, it was not large enough to detect differences in more detailed variables. Further research with a larger sample size would be required to address this issue. Third, the cross-sectional design of this study does not allow for the establishment of causal relationships between independent variables and the primary outcome. The findings can only suggest an association. Finally, the results presented in this study do not directly represent all high-IQ individuals in Indonesia due to the nature of Mensa Indonesia, whose membership predominantly comes from only the central part of the country. Moreover, this study also has limited generalizability to populations in other countries.

## CONCLUSIONS

The prevalence of tobacco smoking among high-IQ individuals in Indonesia is 10.8%, which is significantly lower than the national prevalence. No significant statistical associations were found between sociodemographic data and smoking status in this population. However, there was a tendency for smokers to have a family member who smokes, and individuals with higher annual incomes were less likely to be smokers. Our findings reinforce some existing views that highlight the complexity of association between human intelligence and health impacts.

## Data Availability

The data supporting this research are available from the authors on reasonable request.
